# Immunomodulatory and Antioxidative potentials of adipose-derived Mesenchymal stem cells isolated from breast versus abdominal tissue: a comparative study

**DOI:** 10.1186/s13619-020-00056-2

**Published:** 2020-10-06

**Authors:** Nourhan Abu-Shahba, Marwa Mahmoud, Mazen Abdel-Rasheed, Yasmine Darwish, Ahmad AbdelKhaliq, Eman Mohammed, Mahmoud ElHefnawi, Osama Azmy

**Affiliations:** 1 Stem Cell Research Group, Centre of Excellence for Medical Research, National Research Centre, Cairo, Egypt; 2 Medical Molecular Genetics Department, Human Genetics and Genome Research Division, National Research Centre, Cairo, 12622 Egypt; 3 Department of Reproductive Health Research, Medical Research Division. National Research Centre, Cairo, Egypt; 4 Plastic and Reconstructive Surgery Unit, General Surgery Department, Kasr Al Ainy School of Medicine, Cairo University, Cairo, Egypt; 5 Biomedical Informatics and Chemoinformatics Group, Centre of Excellence for Medical Research, Informatics and Systems Department, National Research Centre, Cairo, Egypt

**Keywords:** Adipose-derived stem cells (ASCs), Breast adipose tissue, Abdominal adipose tissue, Immunomodulatory potential, Antioxidative potential

## Abstract

**Background:**

Adipose-derived stem cells (ASCs) are considered ideal candidates for both research and cellular therapy due to ease of access, large yield, feasibility, and efficacy in preclinical and clinical studies. Unlike the subcutaneous abdominal fat depot, breast ASCs features are still not well recognized, limiting their possible therapeutic use. ASCs were found to exert immunomodulatory and antioxidative activities for maintaining homeostasis and functionality of diseased/damaged tissues. This study aims to investigate the immunomodulatory and antioxidative potentials of breast versus abdominal isolated ASCs to find out which anatomical site provides ASCs with better immunoregulatory and oxidative stress resistance capabilities.

**Methods:**

ASCs were isolated from abdominal and breast tissues. Gene expression analysis was conducted for a panel of immunomodulatory and antioxidative genes, as well as adipokines and proliferation genes. Flow cytometric analysis of a group of immunomodulatory surface proteins was also performed. Finally, the significantly expressed genes have undergone protein-protein interaction and functional enrichment in silico analyses.

**Results:**

Our results revealed similar morphological and phenotypic characteristics for both breast and abdominal ASCs. However, a significant elevation in the expression of two potent immunosuppressive genes, IL-10 and IDO as well as the expression of the multifaceted immunomodulatory adipokine, visfatin, was detected in breast versus abdominal ASCs. Moreover, a significant overexpression of the antioxidative genes, GPX1, SIRT5, and STAT3 and the proliferation marker, Ki67, was also observed in breast ASCs relative to abdominal ones. In silico analysis showed that both of the differentially upregulated immunomodulatory and antioxidative mediators integratively involved in multiple biological processes and pathways indicating their functional association.

**Conclusion:**

Breast ASCs possess superior immunomodulatory and antioxidative capabilities over abdominal ASCs. Our findings shed light on the possible therapeutic applications of breast ASCs in immune-related and oxidative stress-associated diseases.

## Background

Adipose tissue is a connective tissue composed mainly of adipocytes, in addition to preadipocytes, fibroblasts, pericytes, vascular endothelial cells, immune cells, and mesenchymal stem cells (MSCs) (Waki and Tontonoz 2007; Han et al. 2015). Moreover, it acts as a bioactive endocrine organ that secretes soluble factors (Tchkonia et al. 2013). There are two major types of fat: visceral fat and subcutaneous fat (Wronska and Kmiec 2012). Subcutaneous fat represents the largest fat depot in the human body (Abate et al. 1995), containing various subcutaneous fat depots classified according to their anatomical locations. These depots comprise a multipotent mesenchymal stem cell population, known as adipose-derived stem cells (ASCs) (Schipper et al. 2008). ASCs represent one of the most promising stem cell populations due to their ease of access, large yield, being obtained through minimal invasive procedures in addition to their high proliferative and multilineage potentials (Zuk et al. 2001; Locke et al. 2009; O’Halloran et al. 2017). Moreover, ASCs were recommended as good candidates for cellular therapy as they have shown feasibility and efficacy in preclinical and clinical studies (O’Halloran et al. 2017; Gimble et al. 2007; Mizuno et al. 2012).

Recently, several comparative reports have demonstrated that the biological and functional characteristics of ASCs differ according to the site of harvest (Schipper et al. 2008; Hanson et al. 2013; Russo et al. 2014; Nepali et al. 2018). ASCs harvested from superficial abdominal regions exerted higher antiapoptotic action than those harvested from the arm, thigh, trochanteric, and deep abdominal depots (Schipper et al. 2008). Additionally, ASCs derived from orbit showed elevated adipogenic and osteogenic differentiation potential and lower chondrogenic potential relative to abdominal ASCs (Nepali et al. 2018), while the amount of ASCs harvested from abdomen seemed to be higher than that of the hip and thigh regions (Jurgens et al. 2008). Furthermore, breast-isolated ASCs showed higher expression of FGF2 (fibroblast growth factor 2) (Hanson et al. 2013) and was highly enriched in MSCs expressing SSEA-4 (stage-specific embryonic antigen 4) compared to abdominal ASCs (Maddox et al. 2012).

The subcutaneous abdominal fat depot is nearly the most commonly used depot for ASCs harvesting due to its abundance in patients (Schipper et al. 2008; Jurgens et al. 2008; Padoin et al. 2008). However, other fat depots are emerging as possible alternative sources for ASCs (Nepali et al. 2018; Maddox et al. 2012; Rezai Rad et al. 2017) such as breast. Breast fat depot is one of the recently identified ASCs sources; however, little concern has been given to this source. Breast ASCs characteristics are still not clear, limiting their possible therapeutic use (Yang et al. 2014).

ASCs were found to exert several paracrine actions that are essential for homeostatic restoration and proper functionality in diseased/damaged tissues, including the immunomodulatory and antioxidative actions (Liang et al. 2014; Kim et al. 2015; Orgun and Mizuno 2017). The immunomodulatory potential is mediated through secretion of a variety of cytokines which impact different immune cells activities (Leto Barone et al. 2013). This immunoregulatory role had found application in both preclinical and clinical studies of different immune-related diseases such as rheumatoid arthritis, Crohn’s disease, graft versus host disease, and diabetes (Maria et al. 2016). ASCs antioxidative action acts against the accumulation of the reactive oxygen species (ROS) that induce various diseases (Liang et al. 2014), such as neurodegenerative, cardiovascular diseases, and endometriosis (Uttara et al. 2009; Scutiero et al. 2017). Moreover, ASCs were found to alleviate inflammation in the injured areas through both their antioxidative and anti-inflammatory properties (Kim et al. 2015).

Identifying the influence of different fat depots on ASCs characteristics could be essential for developing new cell-based therapeutic strategies. This study aimed to compare between ASCs isolated from two different anatomical sites, to detect which fat depot provides ASCs with more enhanced immunomodulatory and/or antioxidative potential. This would unravel the type of ASCs that would be more suitable for therapeutic application in immune disturbance and/or oxidative stress-induced pathologies.

## Methods

### Isolation of abdominal and breast adipose-derived mesenchymal stem cells

Abdominal adipose tissue samples (*n* = 7) were obtained from subjects undergoing either abdominoplasty or incisional hernia surgeries, while breast adipose tissue samples (n = 7) were obtained from subjects undergoing reduction mammaplasty surgeries after taking informed consent. The study participants aged between 22 and 55 years. The sample collection was ethically approved by the Medical Research Ethics Committee, the National Research Centre, Cairo, Egypt (Registration code: 16–282). Both abdominal and breast ASCs were isolated according to the previously described protocol of Bunnel et al. (Bunnell et al. 2008) with some modifications. The excised fat was dissected using sterile blades and washed in phosphate-buffered saline (PBS, Lonza, Belgium) and then digested with 1 mg/ml type 1A collagenase (Gibco Life Technologies, USA). The digested tissue underwent centrifugation, resuspension in PBS, and filtration through 100 μm nylon mesh cell strainer (Greiner Bio-One, Germany). The cell filtrate was centrifuged, resuspended, and cultured in complete growth media comprising Dulbecco’s Modified Eagle Medium (DMEM, Lonza, USA) supplemented with 10% fetal bovine serum (FBS, Lonza, Belgium), 2% penicillin/streptomycin/amphotericin (Lonza, USA), and 1% Glutamax (Gibco, Life Technologies, USA). Cultured cells were incubated in a 5% CO_2_ humidified atmosphere at 37 °C. Non-adherent cells were removed with the first media exchange. Media exchange was done twice a week and cells were passaged at a 75–80% confluence.

### Morphological identification of isolated ASCs

The typical spindle fibroblast like morphology and plastic adherence features of MSCs were examined using an inverted microscope (Leica Microsystems DMi1, Switzerland) equipped with an imaging system (Leica DMS300, Switzerland). Cell morphology and expansion were followed up and photographed through different passages.

### Characterization of ASCs by flow cytometry

The expression of ASCs characteristic surface markers was analyzed by flow cytometry technique, for abdominal and breast ASCs, as previously described (Fathi and Farahzadi 2018; Fathi et al. 2019) with some modifications. At the second or third passage, a total of 5 × 10^4^ cells were harvested, enzymatically using 0.25% Trypsin-EDTA (Lonza) or manually using a cell scraper, centrifuged at 1600 rpm, and then washed with PBS (Lonza). Cells were incubated for 20–30 min in the dark with the suitable volumes of the following fluorochrome-labeled monoclonal antibodies: CD90-FITC, CD105-PE, CD44-PE, and CD34-FITC (BD, Biosciences, USA). After incubation, another washing step was done using PBS to remove excess stain. Flow cytometric analysis was performed using a Coulter EPICS device (Beckman Coulter, USA) and data analysis was carried out using System II Software.

### Real time PCR gene expression analysis

Total RNA was isolated from abdominal and breast ASCs at the second or third passage using the miRNeasy Mini Kit for efficient RNA isolation (Qiagen, USA) following the manufacturer’s instructions. Briefly, 1.5 × 10^6^ cells were homogenized in QIAzole reagent (Qiagen, USA), mixed with chloroform, and then centrifuged. The upper aqueous phase was obtained and mixed with absolute alcohol and then transferred into the kit separation column in which several washing steps were performed using the kit reagents. Finally, RNA was dissolved in RNase-free water. RNA quantification was performed using a NanoDrop 2000 spectrophotometer (Thermo Fisher Scientific, USA). cDNA was synthesized from 1 μg of extracted RNA using Revert Aid First Strand cDNA Synthesis Kit (Thermo Fisher Scientific, Lithuania). TaqMan gene expression assays (Thermo Fisher Scientific, USA) for the following immunomodulatory mediators: interleukin 10 (IL-10), indoleamine-2,3-dioxygenase (IDO), interleukin 6 (IL-6), tumor necrosis factor (TNF), and TNFα-stimulated gene 6 (TSG-6), and the following adipokines: leptin, resistin, adiponectin, and visfatin, were used as instructed by the manufacturer. Beta-actin was used as a housekeeping gene for normalization (Table 1A).

**Table 1:** (A) Gene expression TaqMan probes (Thermo Fisher Scientific, USA) and (B) predesigned QuantiTect primer assays (Qiagen, USA)

(A) **Taqman Probes used for real time-PCR assay**	
**Probes**	**Taqman transcript assay accession number**
interlukin-10 (IL-10)	Hs00961622_m1
indoleamine-2,3-dioxygenase (IDO)	Hs00984148_m1
interlukin-6 (IL-6)	Hs00174131_m1
tumor necrosis factor (TNF)	Hs00174128_m1
TNFα-stimulated gene 6 (TSG6)	Hs00200180_m1
leptin	Hs00174877_m1
adiponectin	Hs00605917_m1
resistin,	Hs00220767_m1
visfatin	Hs00237184_m1
b-actin	Hs01060665_g1
(B) **Quantitect human primer assays used for real time-PCR**	
**Primer assays**	**Accession Number**
superoxide dismutase 1 (SOD1)	(QT01671551)
superoxide dismutase 2 (SOD2)	(QT01008693)
glutathione peroxidase 1(GPX1)	(QT00203392)
catalase (CAT)	(QT00079674)
sirtuin 3 (SIRT3)	(QT00091490)
sirtuin 4 (SIRT4)	(QT00202503)
sirtuin 5 (SIRT5)	(QT00047537)
Ki67 (MKi67)	(QT00014203)
GAPDH	(QT00079247)

QuantiTect SYBR Green Master Mix Kit (Qiagen, USA) was employed for gene expression analysis of the following antioxidative mediators: superoxide dismutase 1 (SOD1), superoxide dismutase 2 (SOD2), catalase (CAT), glutathione peroxidase 1 (GPX1), sirtuin 3, sirtuin 4, sirtuin 5 (SIRT3–5), and the proliferation marker Ki67/MKI67. QuantiTect predesigned primer assays (Qiagen, USA) for the aforementioned genes were utilized (Table 1B). The following primers were used for the pluripotency and antioxidative transcription factor; STAT3:5′-ATCGAGCAGCTGACTACACTG-3′ and 5′-ATCGAGCAGCTGACTACACTG-3′. Melting point analysis was done to ensure the specificity of the amplification product. GAPDH was used as a housekeeping gene for normalization. Plates were read using the ABI 7500 fast system (Applied Biosystems). The gene expression data were analyzed using the comparative ΔΔCt method (Livak and Schmittgen 2001).

### Flow cytometric analysis of immunoregulatory cell surface proteins

The surface expression of immunoregulatory-related molecules was evaluated for ASCs, at the second or third passage, by flow cytometric analysis using the following fluorochrome-labeled monoclonal antibodies: CD200-PE, CD271-PE, CD274-FITC, and CD276-FITC (BD, Biosciences, USA). Flow cytometry was performed using a Beckman Coulter EPICS XL device (Beckman Coulter, USA) and data analysis was carried out using System II software.

### Bioinformatics analysis

Protein-protein interaction (PPI) and functional enrichment in silico analyses were performed for our significantly upregulated gene set in breast ASCs versus abdominal ASCs (IL-10, IDO, NAMPT, GPX1, SIRT5, STAT3, and ki67/MKI67), using the STRING server, https://string-db.org/ (Szklarczyk et al. 2019), to find out how these genes interact together. Moreover, gene enrichment functional analysis was done to detect the pathways and the biological processes that our genes contribute to. Moreover, functional association gene network prediction analysis was performed using the GeneMANIA server, http://genemania.org/, for the indicated gene set (Franz et al. 2018) to explore the possible gene networks functionally associated with our target genes.

### Statistical analysis

Statistical analysis was carried out using SPSS 16.0 (IBM, New York, USA) for the obtained results by using the independent *t*-test and the Mann–Whitney *U* test. Statistical differences between the abdominal and breast ASCs groups were considered significant at *p*-values less than 0.05.

## Results

### Both breast and abdominal ASCs exhibiting comparative morphological and phenotypic characteristics

**Fig. 1:**
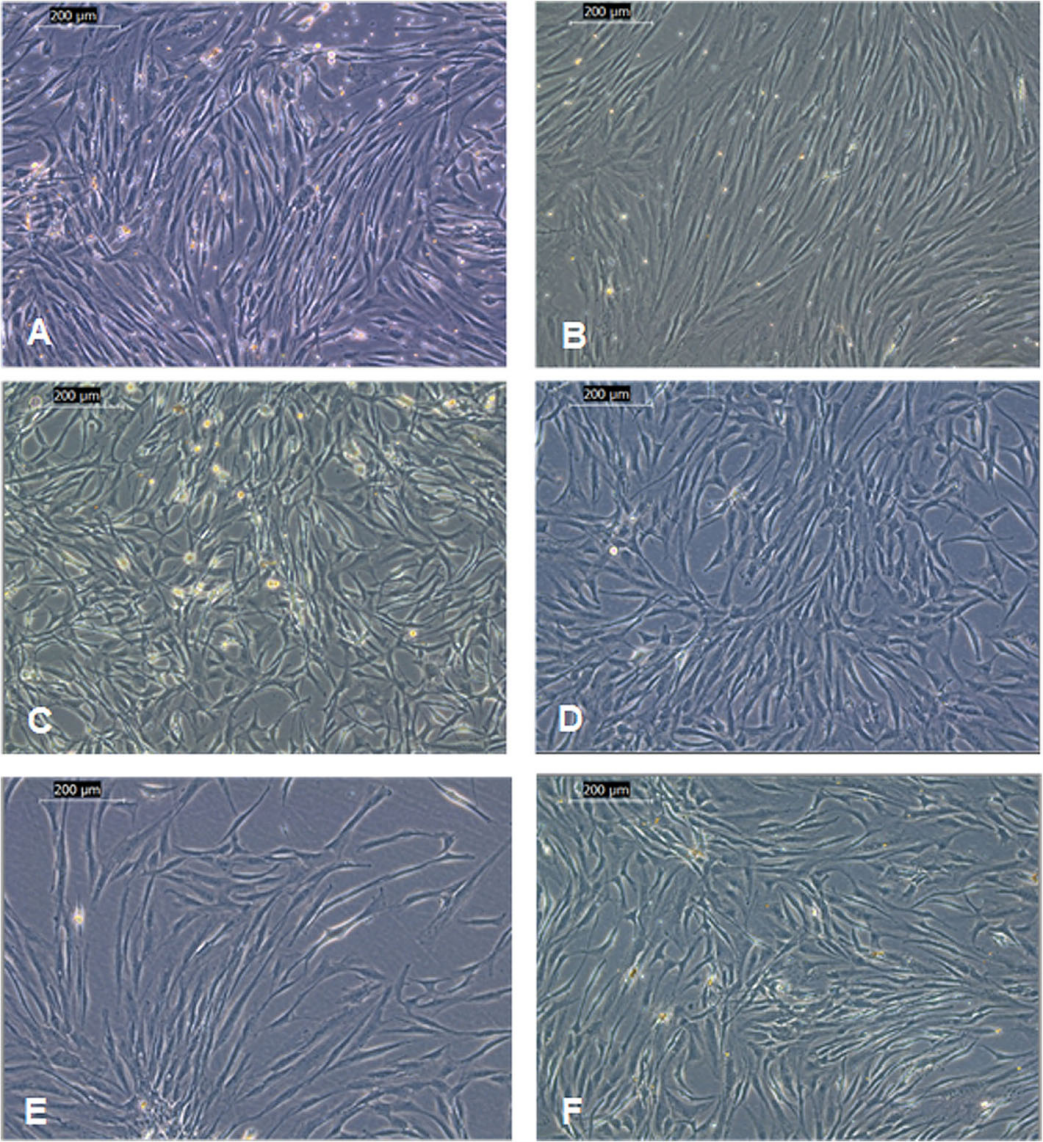
Morphology of abdominal and breast ASCs at different passages. **a**, **c**, and **e** Abdominal ASCs at passages 0, 1, and 2, respectively. **b**, **d**, and **f** Breast ASCs at passages 0, 1, and 2, respectively. Cells of both types of tissues appear with typical MSCs spindle morphology (scale bar = 200 μm)

**Fig. 2:**
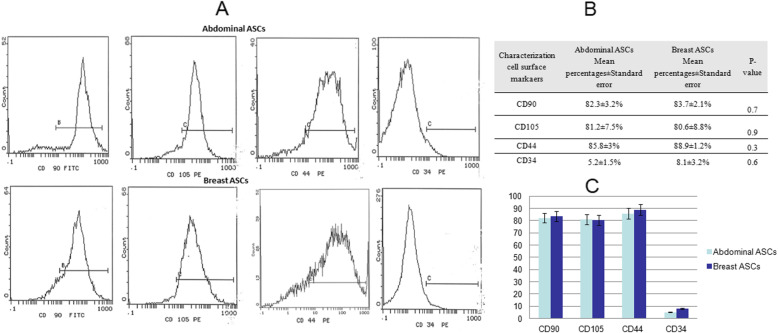
Flow cytometric phenotype analysis for mesenchymal stem cell characteristic surface markers. **a** Flow cytometry charts of CD90, Cd105, CD44, and CD34. **b** Surface expression percentages of each marker along with their *p*-values. **c** A bar chart representing the flow cytometry percentage results. This figure shows high expression of the mesenchymal stem cell markers, CD44, Cd73, CD90, and CD105; low expression of non-mesenchymal surface markers, CD34 exhibited by both abdominal and breast ASCs with no significant differences (*p*-value > 0.05)

The identity of both abdominal and breast ASCs was detected by morphological examination and phenotypic analysis. Both types of ASCs were plastic adherent with typical mesenchymal stem cell morphology as shown under the inverted microscope (Fig. 1). Flow cytometric phenotypic analysis has revealed that ASCs of both abdominal and breast tissues were positive for the MSC characteristic markers, CD90, CD105 and CD44, and negative for the hematopoietic marker, CD34 with no statistical difference between both types of ASCs (*p*-value > 0.05) (Fig. 2).

### Upregulation of immunoregulatory/anti-inflammatory mediators in breast versus abdominal ASCs

**Fig. 3:**
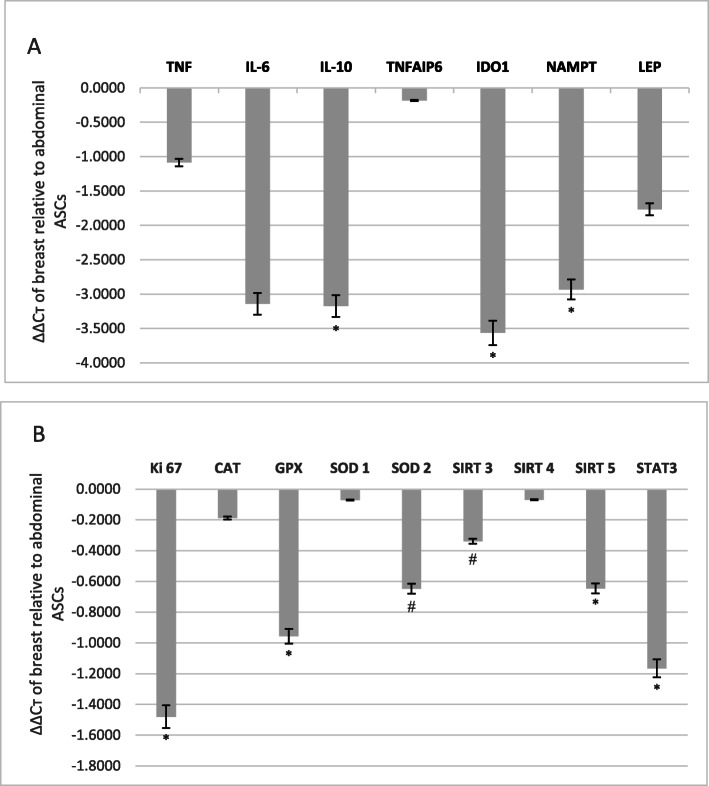
Gene expression levels. **a** Gene expression levels of the immunoregulatory cytokines (TNF, IL-10, IL-6, TSG-6, and IDO) and adipokines (leptin and visfatin) in breast compared to abdominal ASCs represented as ΔΔCт values (* = *p*-value < 0.05). **b** Gene expression levels of the antioxidative markers (SOD1, SOD2, CAT, GPX1, and SIRT3–5), the pluripotency and antioxidative transcription factor (STAT3), and the proliferation marker (Ki67) in breast compared to abdominal ASCs represented as ΔΔCт values (* = *p*-value < 0.05 and # = *p*-value < 0.05 in 5 samples only)

Expression levels of the tested panel of immunomodulatory and adipokine genes have shown upregulation of these genes in breast ASCs compared to abdominal ASCs, some of which were significantly elevated. Two important immunosuppressive genes (IL-10 and IDO) were found significantly elevated in breast ASCs relative to abdominal cells (*p*-values 0.048 and 0.025, respectively). Visfatin adipokine was found also to be significantly upregulated in breast ASCs (*p*-value 0.018) (Fig. 3a). TNF, IL-6, TSG-6, and leptin expression levels were elevated in breast versus abdominal ASCs; however, this elevation did not reach statistical significance. Adiponectin and resistin expression was undetermined in most samples.

### Upregulation of antioxidative, pluripotency, and proliferation markers in breast versus abdominal ASCs

Gene expression analysis also revealed the upregulation of all antioxidative, pluripotency, and proliferation mediators in breast ASCs relative to abdominal ones, with significant elevation in some genes. Breast ASCs constitutively expressed GPX1 (*p*-value 0.003) and SIRT5 (*p*-value 0.013) compared to abdominal ASCs. Expression of SOD2 and SIRT3 was significantly upregulated in five out of seven breast ASCs cultures (*p*-value 0.042 for both). The antioxidative enzymes CAT, SOD1 and SIRT4 showed non-significant upregulation in breast ASC populations indicating comparable gene levels. Moreover, significant elevation was detected for the pluripotency and antioxidative transcription factor, STAT3 (*p*-value 0.025), as well as the proliferation marker Ki67 (*p*-value 0.006) that was highly significant in breast compared to abdominal ASCs supporting the observed superior in vitro expansion potential of breast ASCs population (Fig. 3b).

### Comparative expression of immunoregulatory surface proteins in breast and abdominal ASCs

**Fig. 4:**
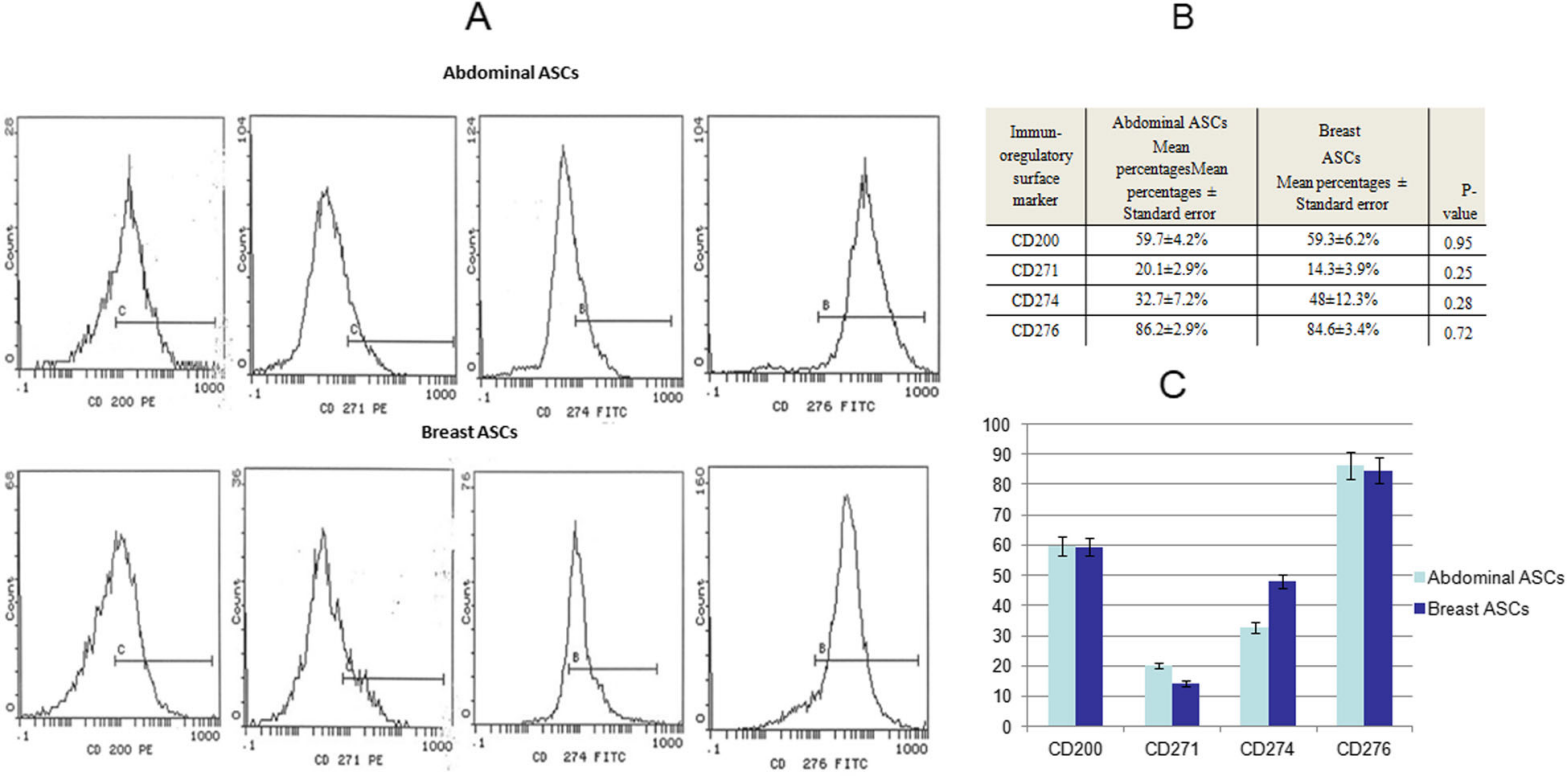
Flow cytometry of abdominal and breast ASCs for surface expression of immunoregulatory surface markers. **a** Flow cytometry charts of CD200, CD271, CD274, and CD276. **b** Surface expression percentages of each marker along with their *p*-values. **c** A bar chart representing the flow cytometry percentage results. Comparable surface expression percentages were observed for both abdominal and breast ASCs as there were no significant differences (*p*-value > 0.05)

Flow cytometric cell surface expression of immunoregulatory surface molecules has shown comparable results for the tested surface molecules in abdominal and breast ASCs where no significant difference was detected (*p*-value > 0.05). The mean expression percentage of CD200 was 59.7 ± 4.2% in abdominal ASCs and 59.3 ± 6.2% in breast ASCs; CD271 expression was 20.1 ± 2.9% in abdominal ASCs and 14.3 ± 3.9% in breast CD274 was 32.7 ± 7.2% in abdominal ASCs and 48 ± 12.3% in breast ASCs; CD276 was 86.2 ± 2.9% in abdominal ASCs and 84.6 ± 3.4% in breast ASCs (Fig. 4).

### Significantly expressed immunomodulatory and antioxidative mediators in breast ASCs acting integratively in various vital biological processes and pathways

**Fig. 5:**
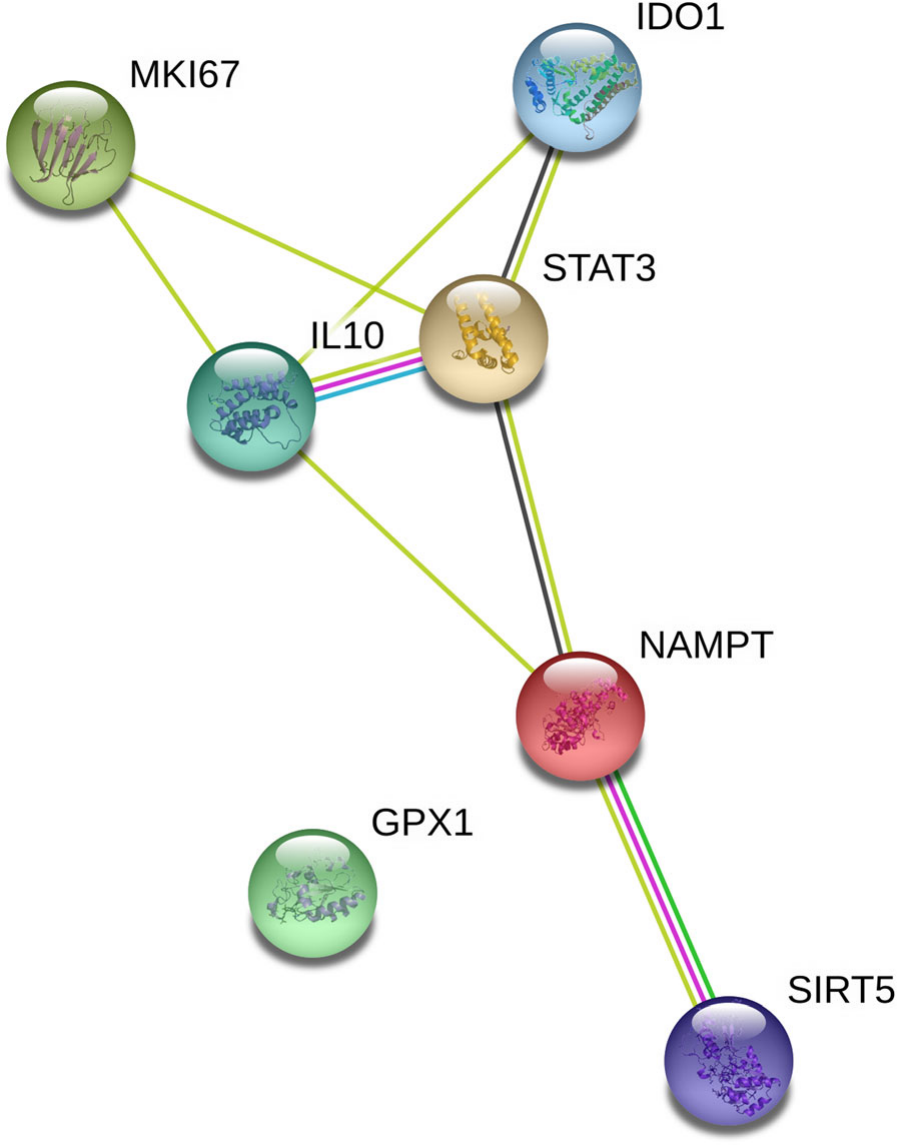
Protein interaction network for the differentially upregulated gene set in breast ASCs compared to abdominal ASCs as obtained by the STRING database. It shows patterns of interaction between IL-10 and STAT3 [experimental (pink line), database annotation (blue line), and automated text-mining (green line)]; NAMPT and SIRT5 [experimental (pink line), automated text-mining (light-green line), and predicted gene neighborhood (dark green line)]; IDO and STAT3 [coexpression (black line) and automated text-mining (light-green line)]; STAT3 and NAMPT [coexpression (black line) and automated text-mining (light-green line)]; IL-10 and NAMPT; IDO and IL-10; STAT3 and Ki67/MKi67; and IL-10 and Ki67/MKi67 [automated text-mining only (light-green line)]

PPI and functional enrichment analysis were performed using the STRING server for our differentially upregulated genes in breast versus abdominal ASCs. It was demonstrated shown that members of this set exhibited a significant PPI enrichment with *p*-value of 0.0008.7 (Fig. 5). A strongly evidenced interaction between IL-10 and STAT3 was indicated by experimental, database annotation, and automated text-mining evidence. Also, NAMPT and SIRT5 showed another strongly evidenced interaction as indicated by experimental and automated text-mining and predicted gene neighborhood evidence. IDO and STAT3 interaction, as well as STAT3 and NAMPT interaction were evidenced by coexpression and automated text-mining. IL-10 and NAMPT and IDO and IL-10 together with IL-10 and Ki67 interactions were proven by automated text-mining only.

Enrichment analysis of our gene set has revealed several significant biological processes with a false discovery rate (FDR) of < 0.01 as shown in Table 2 including the genes contributing to each biological process (Table 2). Moreover, a number of pathways were significantly enriched for our gene set including FoxO signaling pathway (FDR = 0.008), Jak-STAT signaling pathway (FDR = 0.009) (KEGG Pathways), IL-10 (FDR = 0.006), IL-4 and IL-13 signaling pathways (FDR = 0.011) (Reactome Pathways) in which STAT3 and IL-10 are involved.

**Table 2:** Biological processes in which our gene list takes part (FDR < 0.01) as obtained by the STRING database

#term ID	Biological Process	FDR	Matching proteins in the network
GO:2000378	negative regulation of reactive oxygen species metabolic process	0.0007	IL-10, SIRT5, STAT3
GO:0002676	regulation of chronic inflammatory response	0.0017	IDO1, IL-10
GO:0043066	negative regulation of apoptotic process	0.0017	GPX1, IDO1, IL-10, SIRT5, STAT3
GO:0002862	negative regulation of inflammatory response to antigenic stimulus	0.0018	GPX1, IL-10
GO:0070230	positive regulation of lymphocyte apoptotic process	0.0035	IDO1, IL-10
GO:1903427	negative regulation of reactive oxygen species biosynthetic process	0.0038	IL-10, STAT3
GO:0008283	cell population proliferation	0.0045	GPX1, IL-10, MKI67, STAT3
GO:0001659	temperature homeostasis	0.0046	GPX1, STAT3
GO:0042127	regulation of cell population proliferation	0.0046	GPX1, IDO1, IL-10, NAMPT, STAT3
GO:1903202	negative regulation of oxidative stress-induced cell death	0.0062	GPX1, IL-10
GO:0050678	regulation of epithelial cell proliferation	0.0064	GPX1, IL-10, STAT3
GO:0032655	regulation of interleukin-12 production	0.0072	IDO1, IL-10
GO:0042130	negative regulation of T cell proliferation	0.0075	IDO1, IL-10
GO:0050727	regulation of inflammatory response	0.0075	GPX1, IDO1, IL-10

*GO* Gene ontology, *FDR* False discovery rate

**Fig. 6:**
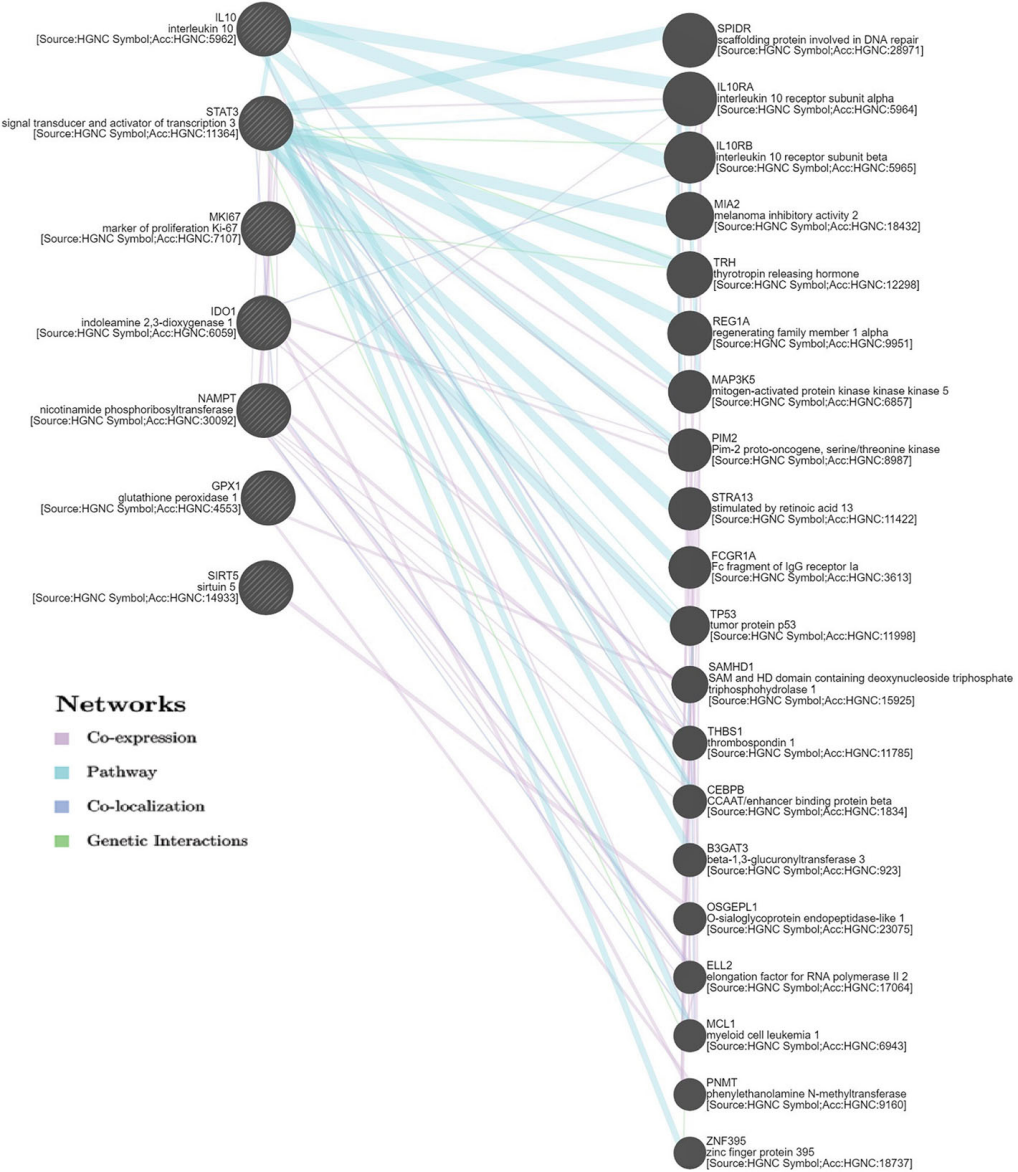
Functional association gene network as obtained by GeneMANIA server for the significantly upregulated genes in breast ASCs versus abdominal ones. This network includes twenty functionally associated genes that are coexpressed, colocalized, genetically interact, or share the same pathway with our gene set

Functional association gene network prediction was performed using the GeneMANIA server for our differentially upregulated genes in breast versus abdominal ASCs. It provided 20 functionally associated genes with our gene set; these functional associations included coexpression, colocalization, genetic interaction, and sharing common pathways. The 20 genes are demonstrated in Fig. 6 (Supplementary Table 1).

## Discussion

Since the physiological microenvironment of fat depots differs from one anatomical site to another, the biological and functional characteristics of tissue-resident ASCs may vary accordingly. Up to our knowledge, the current study is one of the first comparative studies addressing the immunomodulatory potential in breast versus abdominal ASCs and the first study to compare their antioxidative potentials.

Previously, it has been shown that ASCs characteristics are based on inherent properties as well as microenvironmental factors that vary according to the anatomical region of the adipose tissue (Fuchs et al. 2004; Guneta et al. 2016). Few studies adressing the coparison between breast and abdominal ASCs have been conducted. Some of these studies revealed higher self-renewal capability (Guneta et al. 2016), significant overexpression of FGF2 (Hanson et al. 2013), and higher enrichment in MSCs subpopulation SSEA-4 (Maddox et al. 2012). Meanwhile, some studies have reported similarities between the two types of ASCs in some features such as cell surface phenotype and multilineage differentiation capabilities (Hanson et al. 2013; Maddox et al. 2012; Kim et al. 2013; Choudhery et al. 2015), growth kinetics (Choudhery et al. 2015), impact on macrophage gene expression when cocultured together, and enhancing effect on breast cancer cell line proliferation (Kim et al. 2013). On the other hand, Kim et al. (Kim et al. 2013) reported significant upregulation of the proinflammatory cytokine, IL-1B in abdominal ASCs compared to breast ASCs, while Guneta et al. (Guneta et al. 2016) reported that abdominal ASCs may be better than breast ASCs in cell-assisted fat grafting and adipose tissue turnover, as they demonstrated a higher tendency to adipogenic differentiation in abdominal ASCs than breast-isolated ones.

Using the flow cytometric immunophenotype analysis, the identity of our isolated cells was confirmed to be mesenchymal in origin (non-hematopoietic), where our isolated cells expressed the mesenchymal stem cell markers CD90, CD105, and CD44 with high percentages, while showing low expression of the hematopoietic marker, CD34. In accordance with previous reports (Hanson et al. 2013; Maddox et al. 2012; Guneta et al. 2016; Kim et al. 2013; Choudhery et al. 2015), our study revealed that both breast and abdominal ASCs display MSC characteristic morphology and phenotypic cell surface markers with no significant differences (*p*-value > 0.05) in terms of the expression in both types. Additionally, there was no significant difference in the expression of the investigated immunoregulatory surface markers (CD200, Cd271, CD274, and CD276) between abdominal and breast ASCs (*p*-value > 0.05), indicating that both types of ASCs may have similar direct cell-to-cell immunomodulatory ability.

Investigating the immunomodulatory potential revealed significant overexpression of two potent anti-inflammatory mediators, IL-10 and IDO, in breast compared to abdominal ASCs. As previously reported, the immune action demands a balance between pro- and anti-inflammatory responses to accomplish an effective defense against infection while preventing immune-induced tissue damage. IL-10 and IDO are two major immunoregulatory cytokines through which MSCs contribute to achieving this balance (Couper et al. 2008; Kyurkchiev et al. 2014). IL-10 is considered as a master immune regulator (Couper et al. 2008; Iyer and Cheng 2012) playing a major anti-inflammatory role, thereby preventing tissue damage, induced by inflammatory and autoimmune pathologies. IL-10 contributes to inhibition of proinflammatory cytokine secretion (Kyurkchiev et al. 2014). Our gene expression result for IL-10 is in agreement with that of Kim et al. (Kim et al. 2013) comparative study, who reported also a higher expression for IL-10 in breast ASCs compared to abdominal ASCs. However, their study did not report statistical significance, possibly due to the difference in sample size between the two studies (Kim et al. 2013). IDO is a tryptophan-catabolizing enzyme that is one of the key immunoregulators secreted by MSCs. It mediates the immunosuppressive role of MSCs through catalyzing the degradation of tryptophan to N-formylkynurenine in different immune cells (Kyurkchiev et al. 2014; Chen et al. 2011). IDO inhibits the proliferation and differentiation of T cells, while stimulating T regulatory cells to exert their suppressive action (Kyurkchiev et al. 2014; Sharma et al. 2009). Collectively, our findings indicate the superior immunomodulatory potential of breast-derived ASCs over the abdominal ones which may render these cells as attractive tools for therapeutic use in immune-related and inflammatory diseases such as diabetes, nephritis as well as autoimmune diseases, especially in women.

The current study has also revealed a significant upregulation in the expression of the fat-secreted protein, visfatin, in breast compared to abdominal ASCs. Visfatin adipokine is also known as pre-B cell colony-enhancing factor (PBEF) (Tilg and Moschen 2008; Skoczylas 2009) and cytosolic nicotinamide phosphoribosyltransferase where it takes part in nicotinamide adenine dinucleotide (NAD+) biosynthesis during glucose and lipid metabolism (Skoczylas 2009; Coelho et al. 2013). Visfatin is a multifaceted adipokine that plays various roles in multiple processes such as metabolism, inflammation, cell proliferation, and obesity, as well as having different effects on various diseases. Visfatin exerts hypoglycemic effects by increasing glucose uptake by peripheral tissue and inhibiting its release from the liver (Tilg and Moschen 2008; Kang and Cha 2011; Lee et al. 2015). Importantly, visfatin has been reported to act as an immunomodulatory cytokine (Tilg and Moschen 2008; Garten et al. 2009) playing a dual regulatory role during the inflammation process (Xiao et al. 2015). Therefore, studying the factors that direct the immunomodulatory behavior of visfatin in ASCs is recommended.

Gene expression analysis of TNF, IL-6, and TSG-6 and leptin adipokine demonstrated comparable levels in breast ASCs versus abdominal ones, where there was no statistically significant difference. Our result for TNF and IL-6 expression was similar to that of Kim et al. (Kim et al. 2013), who reported also a statistical similarity in their expression.

To the best of our knowledge, no previous studies compared the antioxidative potentials of breast versus abdominal ASCs. In the current study, the antioxidative capacity of breast versus abdominal ASCs was investigated and our findings demonstrated that breast ASCs adopt an enhanced antioxidative defense mechanism. Significant differences between both ASC populations were found in the expression of a number of antioxidant genes including GPX1, SIRT5, and STAT3. GPX1 was the most significantly upregulated antioxidative gene (*p*-value 0.003). GPX1 is a major member of the GPX family in terms of modulating cellular oxidant stress responses and the regulation of stem cells (Lubos et al. 2011; Jiao et al. 2017). It was previously reported that, by detoxifying hydrogen peroxide and fatty acid hydroperoxides, GPX1 is overexpressed, decreasing the oxidative mitochondrial DNA damage (Legault et al. 2000) and enhancing resistance to oxidant-induced apoptotic cell death (Lubos et al. 2011). SIRT5 is the major mitochondrial desuccinylase (Du et al. 2011) and has been shown to activate SOD1 (Lin et al. 2013). Based on its known antioxidative functions, it is hypothesized that SIRT5 helps in maintaining ROS at low levels to preserve stem cell function and longevity (Denu and Hematti 2016). The survival-promoting transcription factor STAT3 has been shown to exert an antioxidative action (Barry et al. 2009). Activation of STAT3 by different stimuli has been proved to protect ASCs from oxidative stress and enhance their therapeutic potency (Liu et al. 2014; Han et al. 2017). Thus, the significantly elevated upregulation of GPX1, SIRT5, and STAT3 reported here in breast ASC population suggests the possible application of these cells in oxidative stress-linked diseases such as cardiovascular and neurodegenerative diseases, endometriosis, and other gynecological conditions.

A number of studies have monitored the expression of antioxidant defense genes in MSCs at baseline. Valle-Prieto and Conget (Valle-Prieto and Conget 2010) reported that bone marrow MSCs express comparable levels of active forms of CAT, GPX, and SOD; however, others demonstrated that MSCs had lower basal antioxidant activity compared to more differentiated cell types (Orciani et al. 2010; Ko et al. 2011). A recent study, which profiled the expression of pro- and antioxidant genes in human visceral versus subcutaneous ASCs, reported upregulation of genes implicated in antioxidative activities including XDH and GPX3 and CAT and HMOX1, in most subcutaneous ASC cultures, while oxidative stress-inducing genes such as NOX1, NOX2, NOX3, and NOS3 were higher in visceral ASCs. This indicates the fat depot-specific molecular differences in pro- and antioxidant genes that may underlie depot-specific oxidative stress levels and associated-cell functions (Sriram et al. 2019).

In the current study, SOD2 and SIRT3 were found significantly expressed in most breast ASC samples over abdominal ones. One of the primary functions of SOD2 is to protect mitochondrial DNA against oxidative damage and to keep mitochondrial functions and reliability (Sun et al. 2015; Matsuda et al. 2018). Additionally, Nrf-2 mediated expression of SOD2, among others, was found to protect MSCs from oxidative stress-induced apoptosis and cytotoxicity (Mohammadzadeh et al. 2012). Besides, SIRT3 is the major mitochondrial deacetylase involved in reducing oxidative stress (Denu 2017; Singh et al. 2018). Significantly elevated expression of both SOD2 and SIRT3 of breast ASCs in this study confirms our findings of the superior antioxidative potential of this cell population over abdominal ASCs. Thus, enhanced mitochondrial functions and integrity may be anticipated to be in breast compared to abdominal ASCs. However, this significance was detected in five samples only; therefore, larger sample size and SOD2 activity assessment are warranted to confirm these results.

Taken together, our observation of augmented immunomodulatory and antioxidative capacities in breast ASCs over abdominal ASCs may be attributed to the unique dynamic mammary microenvironment and the supportive role that adipose tissue plays, providing endocrine mediators and regulatory signals, throughout the different changes in mammary tissue (Wang et al. 2010; Zwick et al. 2018). Our findings suggest that ASCs contribute to maintaining the immune homeostasis and resisting oxidative stress during the different mammary remodeling and involution cycles and hence preventing any abnormal physiological changes or tumor development.

Ki67 or MKI67 gene expression analysis has shown significant upregulation in breast ASCs compared to abdominal ones. This finding may possibly indicate an enhanced proliferation capacity as Ki67 is a marker associated with cell proliferation and is expressed in different cell cycle phases except for G0 phase (Alicka et al. 2019). This conclusion was supported by Guneta et al.’s (Guneta et al. 2016) findings who compared breast and abdominal fat-derived MSCs in terms of proliferation and clonogenic potential. They reported a significantly higher colony-forming ability and self-renewal potentials for breast ASCs than their abdominal counterparts. In their research, the population doubling analysis of both ASC populations demonstrated that breast fat-derived MSCs grew faster with every population doubling; however, abdominal ASCs showed more growth stability (Guneta et al. 2016). Enhanced proliferation potential of breast ASCs over their abdominal counterparts may be attributed to their superior potential to scavenge ROS avoiding their detrimental effects on cell survival and proliferation at high levels (Jeong and Cho 2015). Furthermore, the dynamic nature of breast tissue, which undergoes repeated cycles of growth and involution along with the supportive role of breast adipose tissue during these physical changes (Zwick et al. 2018), may explain the observed enhanced proliferation potential and fast growth ability of breast ASCs.

Identifying the experimental and predicted protein-protein interaction networks as well as functionally associated gene networks is essential to understand the cellular processes at the system-level and helps in discovering new aspects for future research (Schwartz et al. 2009). Bioinformatics analysis herein revealed that the members of our significantly upregulated set of genes in breast ASCs versus abdominal ASCs display a high chance of interaction with each other as well as their integrative contribution in several anti-inflammatory/immunoregulatory, antioxidative, and proliferation-related biological processes (Table 2). For example, negative regulation of ROS metabolic process was found to be a significantly enriched biological process (FDR = 0.0007) to which IL-10, SIRT5, and STAT3 contribute. In addition, negative regulation of inflammatory response to the antigenic stimulus was significantly enriched (FDR = 0.0018) with the contribution of GPX1 and IL-10. Similarly, the cell population proliferation process was found to be significant (FDR = 0.0045), a process where GPX1, IL-10, Ki67/MKI67, and STAT3 act together. Enrichment analysis also revealed two important signaling pathways: FoxO signaling pathway (FDR = 0.008) and Jak-STAT signaling pathway (FDR = 0.009) which contribute to the self-renewal and regenerative potential of stem cells (Tothova and Gilliland 2007; Lee et al. 2016). Besides, immunosuppressive signaling pathways such as interleukin 10 (FDR = 0.006) and interleukin 4 and interleukin 13 signaling pathways (FDR = 0.011) were also significantly enriched (Kucharzik et al. 1998). This indicates the enhancement of these processes in the breast over abdominal ASCs, supporting our experimental results. In addition, twenty genes were computationally predicted to be functionally associated with our gene set, indicating their possible upregulation in breast ASCs and opening the way for further studies concerning their expression and their role in breast ASCs (Fig. 6, Supplementary Table 1).

## Conclusions

From this study, it can be deduced that both breast and abdominal ASCs exhibit similar morphological and phenotypic characteristics; however, breast ASCs exhibit higher proliferative capability as well as superior immunomodulatory and antioxidative potentials compared to abdominal ASCs. These findings may encourage the therapeutic use of breast ASCs especially in autoimmune, inflammatory, and/or oxidative stress-related disorders such as diabetes, endometriosis, cardiovascular diseases and others. However, further in vitro and in vivo studies are required to investigate the safety and efficiency of this emerging type of ASCs for use in cell-based therapy.

## Supplementary information

**Additional file 1: Supplementary Table 1.** Functional association data as obtained by GENEmania database for our input gene set showing the types of functional association between our genes of interest and twenty associated genes.

## Data Availability

All data generated or analyzed during this study are included in this published article and its supplementary information files.

## Abbreviations

APC: Allophycocyanin
ASCs: Adipose-derived stem cells
CAT: Catalase
CD: Cluster of differentiation
DMEM: Dulbecco’s Modified Eagle Medium
DNA: Deoxyribonucleic acid
FBS: Fetal bovine serum
FDR: False discovery rate
FGF-2: Fibroblast growth factor-2
FITC: Fluorescein isocyanate
FOXO: Forkhead box O
GO: Gene ontology
GPX1: Glutathione peroxidase 1
HMox1: Heme oxygenase (decycling) 1
IDO: Indoleamine-2,3-dioxygenase
IL-6, IL-10: Interleukin-6, Interleukin-10
Jak-STAT: Janus Kinase/Signal Transducer and Activator of Transcription.
MKI67: Marker of proliferation ki67
MSCs: Mesenchymal stem cells
NAD: Nicotinamide adenine dinucleotide.
NOS3: Nitric Oxide Synthase 3
NOX1–3: NADPH oxidase 1–3
NrF-2: Nuclear factor erythroid 2-related factor 2
PBEF: Pre-B cell colony-enhancing factor
PBS: Phosphate buffer saline
PE: Phycoerythrin
PPI: Protein-protein interaction
RNA: Ribonucleic acid
ROS: Reactive oxygen species
SIRT3–5: Sirtuin 3–5
SOD1: Superoxide dismutase 1
SSEA-4: Stage Specific Embryo Antigen-4
STAT3: Signal transducer and activator of transcription 3
TNF: Tumor necrosis factor
TSG-6: TNFα-stimulated gene-6
XDH: Xanthine Dehydrogenase
