# Supplementary material for: Immunomodulatory and Antioxidative potentials of adipose-derived Mesenchymal stem cells isolated from breast versus abdominal tissue: a comparative study
Source: Cell Regen. 2020 Oct 6;9:18. doi: 10.1186/s13619-020-00056-2 (PMC7536259; doi:10.1186/s13619-020-00056-2)
Supplement: Supplementary file 1 — Additional file 1: Supplementary Table 1. Functional association data as obtained by GENEmania database for our input gene set showing the types of functional association between our genes of interest and twenty associated genes. [file 13619_2020_56_MOESM1_ESM.docx]

Supplementary table 1: Functional association data as obtained by GENEmania database for our input gene set showing the types of functional association between our genes of interest and twenty associated genes.

| Gene 1 | Gene 2 | Weight | Functional association |
| --- | --- | --- | --- |
| STAT3 | NAMPT | 0.025674 | Co-expression |
| MAP3K5 | STAT3 | 0.012075 | Co-expression |
| PIM2 | IDO | 0.011752 | Co-expression |
| SAMHD1 | GPX1 | 0.015888 | Co-expression |
| SAMHD1 | IDO | 0.019408 | Co-expression |
| THBS1 | NAMPT | 0.0219 | Co-expression |
| THBS1 | STAT3 | 0.022068 | Co-expression |
| OSGEPL1 | SIRT5 | 0.028301 | Co-expression |
| ELL2 | NAMPT | 0.027069 | Co-expression |
| PNMT | GPX1 | 0.017049 | Co-expression |
| PNMT | IDO | 0.020383 | Co-expression |
| PNMT | PIM2 | 0.010653 | Co-expression |
| PNMT | SAMHD1 | 0.020324 | Co-expression |
| STAT3 | NAMPT | 0.013289 | Co-expression |
| IL10RA | STAT3 | 0.013026 | Co-expression |
| PIM2 | STAT3 | 0.008283 | Co-expression |
| PIM2 | IL10RA | 0.014604 | Co-expression |
| CEBPB | IL10RA | 0.014375 | Co-expression |
| CEBPB | PIM2 | 0.013619 | Co-expression |
| B3GAT3 | STRA13 | 0.00457 | Co-expression |
| ELL2 | PIM2 | 0.01202 | Co-expression |
| MCL1 | IL-10 | 0.01046 | Co-expression |
| MCL1 | IL10RA | 0.011353 | Co-expression |
| MCL1 | PIM2 | 0.014055 | Co-expression |
| MCL1 | CEBPB | 0.013628 | Co-expression |
| MCL1 | ELL2 | 0.010441 | Co-expression |
| STAT3 | NAMPT | 0.013125 | Co-expression |
| PIM2 | IDO | 0.005428 | Co-expression |
| SAMHD1 | IDO | 0.012907 | Co-expression |
| ELL2 | NAMPT | 0.014699 | Co-expression |
| STAT3 | NAMPT | 0.006632 | Co-expression |
| IL10RA | NAMPT | 0.009837 | Co-expression |
| PIM2 | STAT3 | 0.009506 | Co-expression |
| PIM2 | TRH | 0.011918 | Co-expression |
| TP53 | MKI67 | 0.012222 | Co-expression |
| THBS1 | NAMPT | 0.01428 | Co-expression |
| THBS1 | STAT3 | 0.01089 | Co-expression |
| CEBPB | NAMPT | 0.009243 | Co-expression |
| CEBPB | STAT3 | 0.007415 | Co-expression |
| CEBPB | IL10RA | 0.012711 | Co-expression |
| CEBPB | PIM2 | 0.017059 | Co-expression |
| MCL1 | IL10RA | 0.012712 | Co-expression |
| MCL1 | CEBPB | 0.010649 | Co-expression |
| STAT3 | NAMPT | 0.005213 | Co-expression |
| THBS1 | IL-10 | 0.007112 | Co-expression |
| CEBPB | NAMPT | 0.012674 | Co-expression |
| ELL2 | NAMPT | 0.010019 | Co-expression |
| STAT3 | NAMPT | 0.009588 | Co-localization |
| IL10RB | IDO | 0.005549 | Co-localization |
| IL10RB | IL10RA | 0.006675 | Co-localization |
| THBS1 | STAT3 | 0.011552 | Co-localization |
| ELL2 | NAMPT | 0.006853 | Co-localization |
| MCL1 | NAMPT | 0.007225 | Co-localization |
| MCL1 | THBS1 | 0.009363 | Co-localization |
| IDO | MKI67 | 0.001762 | Genetic Interactions |
| IL10RB | STAT3 | 0.0006 | Genetic Interactions |
| TRH | MKI67 | 0.001241 | Genetic Interactions |
| TRH | STAT3 | 0.001529 | Genetic Interactions |
| MCL1 | STAT3 | 0.000643 | Genetic Interactions |
| ZNF395 | PNMT | 0.003102 | Genetic Interactions |
| STAT3 | IL-10 | 0.028393 | Pathway |
| SPIDR | STAT3 | 0.106879 | Pathway |
| IL10RA | IL-10 | 0.106306 | Pathway |
| IL10RA | STAT3 | 0.007993 | Pathway |
| IL10RB | IL-10 | 0.122138 | Pathway |
| IL10RB | IL10RA | 0.034385 | Pathway |
| MIA2 | STAT3 | 0.099517 | Pathway |
| TRH | STAT3 | 0.101665 | Pathway |
| REG1A | STAT3 | 0.098408 | Pathway |
| MAP3K5 | IL-10 | 0.083511 | Pathway |
| MAP3K5 | IL10RA | 0.023511 | Pathway |
| MAP3K5 | IL10RB | 0.027012 | Pathway |
| PIM2 | STAT3 | 0.069868 | Pathway |
| STRA13 | STAT3 | 0.091649 | Pathway |
| FCGR1A | STAT3 | 0.067397 | Pathway |
| TP53 | MKI67 | 0.110831 | Pathway |
| TP53 | STAT3 | 0.001355 | Pathway |
| THBS1 | TP53 | 0.00647 | Pathway |
| CEBPB | IL-10 | 0.050935 | Pathway |
| CEBPB | STAT3 | 0.00383 | Pathway |
| B3GAT3 | STAT3 | 0.058164 | Pathway |
| MCL1 | STAT3 | 0.046375 | Pathway |
| ZNF395 | STAT3 | 0.046914 | Pathway |
